# Novel Technologies for Assessing Dietary Intake: Evaluating the Usability of a Mobile Telephone Food Record Among Adults and Adolescents

**DOI:** 10.2196/jmir.1967

**Published:** 2012-04-13

**Authors:** Bethany L Daugherty, TusaRebecca E Schap, Reynolette Ettienne-Gittens, Fengqing M Zhu, Marc Bosch, Edward J Delp, David S Ebert, Deborah A Kerr, Carol J Boushey

**Affiliations:** ^1^Department of NutritionPurdue UniversityWest Lafayatte, INUnited States; ^2^Epidemiology ProgramUniversity of Hawaii Cancer CenterHonolulu, HIUnited States; ^3^School of Electrical and Computer EngineeringPurdue UniversityWest Lafayette, INUnited States; ^4^School of Public HealthCurtin UniversityPerthAustralia

**Keywords:** Mobile telephone food record, dietary assessment, technology, image analysis, volume estimation

## Abstract

**Background:**

The development of a mobile telephone food record has the potential to ameliorate much of the burden associated with current methods of dietary assessment. When using the mobile telephone food record, respondents capture an image of their foods and beverages before and after eating. Methods of image analysis and volume estimation allow for automatic identification and volume estimation of foods. To obtain a suitable image, all foods and beverages and a fiducial marker must be included in the image.

**Objective:**

To evaluate a defined set of skills among adolescents and adults when using the mobile telephone food record to capture images and to compare the perceptions and preferences between adults and adolescents regarding their use of the mobile telephone food record.

**Methods:**

We recruited 135 volunteers (78 adolescents, 57 adults) to use the mobile telephone food record for one or two meals under controlled conditions. Volunteers received instruction for using the mobile telephone food record prior to their first meal, captured images of foods and beverages before and after eating, and participated in a feedback session. We used chi-square for comparisons of the set of skills, preferences, and perceptions between the adults and adolescents, and McNemar test for comparisons within the adolescents and adults.

**Results:**

Adults were more likely than adolescents to include all foods and beverages in the before and after images, but both age groups had difficulty including the entire fiducial marker. Compared with adolescents, significantly more adults had to capture more than one image before (38% vs 58%, *P *= .03) and after (25% vs 50%, *P * = .008) meal session 1 to obtain a suitable image. Despite being less efficient when using the mobile telephone food record, adults were more likely than adolescents to perceive remembering to capture images as easy (*P * < .001).

**Conclusions:**

A majority of both age groups were able to follow the defined set of skills; however, adults were less efficient when using the mobile telephone food record. Additional interactive training will likely be necessary for all users to provide extra practice in capturing images before entering a free-living situation. These results will inform age-specific development of the mobile telephone food record that may translate to a more accurate method of dietary assessment.

## Introduction

Dietary intake is an important environmental exposure to consider when evaluating an individual’s or population’s risk for chronic disease. A link between diet and the development of certain cancers, cardiovascular disease, liver disease, and type 2 diabetes has been established. However, scientific evidence linking diet and genetics to these diseases continues to emerge [1]. The development of genome-wide association studies has led to the identification of genetic variations associated with risk for diseases such as type 2 diabetes [2], atherosclerosis [3], and Crohn disease [4]. Diet and genetics may play a shared role in the etiology of or protection from many diseases. Methodological issues with dietary assessment, however, have limited the ability to identify gene–nutrient interactions.

Dietary assessment is difficult due to the increasing complexity of the food supply and day-to-day variability in a person’s diet [5]. Traditional self-report methods of dietary assessment, including the 24-hour dietary recall, food record, and food frequency questionnaire [6], rely on the respondent’s memory and ability to estimate portion sizes. Both adults and adolescents tend to underreport total energy intake by as much as 30% [7-12]. Developing diet assessment methods that can be incorporated into the lifestyle of adolescents is especially difficult. Adolescents are in a rapid phase of growth requiring increased energy, eat more frequently, and have more unstructured eating events outside of the home [13]. There is much day-to-day variability in the composition and timing of their eating occasions, leading to forgetfulness and lack of compliance in recording their dietary intake [14]. Adolescents also report becoming irritated with their parents reminding them to complete their food records [15]. Adults, on the other hand, follow a more regular routine than adolescents. Senior adults may have more consistent meal times, while working adults may be more influenced by the demands and characteristics of their working environment. However, all adults may encounter occasions where their more structured routines are disrupted by events that make accurate recording via the current assessment methods more difficult. In addition to being burdensome to the respondent, these methods can be expensive and labor intensive for the researcher. The Genes, Environment and Health Initiative of the National Institutes of Health in the United States is attempting to address many of these shortcomings by supporting the development of novel methods to assess diet and of high-throughput methods to assess genetic profiles in individuals and populations [16].

Researchers have been striving to harness the potential of new digital technologies to improve the effectiveness of their work, and researchers in the field of dietary assessment are no different. The past 10 to 15 years has seen steadily increasing usage of mobile communication devices [17]. Significant advances in the capabilities of these devices have coincided with mobile phones achieving the status of an essential communication tool, so that mobile computing devices, such as mobile telephones with cameras known as smart phones, are now poised to realize their potential as a computing device with specific health applications. Personal digital assistants (PDAs) were the first generation of mobile computers used for data collection [18,19]. However, some of the initial studies using PDAs were not promising [20], as earlier PDAs used technology that lacked user-friendly options, and backlit screens made their content difficult to see. As a result of these limitations, early investigators concluded that the technology was a barrier to collecting accurate information.

However, with the rapid advancement in the capabilities of mobile devices, researchers are now pursuing image-based methods as a way of addressing the limitations of traditional dietary assessment methods [21-23]. The use of mobile applications to assist in the monitoring of diabetes, physical activity, and smoking cessation has previously been discussed in the literature [24-26] and has informed the use of these tools for new diet assessment methods. The development of a mobile telephone food record for adults and adolescents for use in a new, image-based dietary assessment method, partially supported by the Genes, Environment and Health Initiative, was the subject of this study.

The design of the mobile telephone food record has been described previously [27]. For all users, the task of recording images of their food should be relatively quick and easy for it to be acceptable. Briefly, participants would use the mobile telephone food record application to capture images of their foods and beverages before and after eating. Methods of image analysis [28,29] are used to automatically identify the food in the image. With the inclusion of a fiducial marker, an object of known dimension and size, the volume of consumption can be estimated. The information from image analysis and volume estimation can be linked to a nutrient database to compute the energy and nutrients consumed, so this method will not have to rely on the respondent’s memory and ability to estimate portion sizes. Additionally, real-time data collection eliminates the need for researchers to enter and code food records. Ideally, the ease of use of mobile telephone food record will result in an accurate dietary assessment tool for both adults and adolescents.

There are challenges related to using smart phones in this new dietary assessment method. For example, for adolescents to use the device, school administrators must accept its use on the school campus, as young people are in school most days of the week. Adults are often less facile than adolescents with using new technology. Therefore, the mobile telephone food record design needs to address these concerns.

Evidence-based development is a crucial step in designing the mobile telephone food record for use by both adults and adolescents [30]. The form of evidence-based development of the mobile telephone food record is an interaction design, which is the discipline of defining the characteristics of products that a user can interact with in their everyday and working lives [30]. The mobile telephone food record design process, when applying interaction design, is an iterative cycle of usability testing in which the user feedback is applied to the next version of the mobile telephone food record, which is tested again [27]. Using this process has allowed the design of the mobile telephone food record to evolve from the perspective of the user or client, resulting in a more positive experience for the user.

The objectives of this study were to evaluate a defined set of user skills for both adults and adolescents—that is, successful image capturing of an eating occasion, while using the mobile telephone food record—and to compare the perceptions and preferences between adults and adolescents regarding their use of the mobile telephone food record. A priori, our hypothesis was that statistically significant differences between adults and adolescents would emerge that would need to be translated into different mobile telephone food record designs to accommodate lifestyles and abilities to use a new technology.

## Methods

### Study Design and Participant Recruitment

We collected data from two samples of adolescent participants [27] and one sample of adult participants. The data collected from the adolescent samples are combined in this analysis (n = 78). The study methods for all samples were approved by the Purdue University Institutional Review Board. Informed assent and consent were obtained from the adolescent participants and their parents, respectively. The adults completed informed consent prior to participation.

The first adolescent sample was drawn from summer camps for adolescents, ages 11–18 years, taking place on the campus of Purdue University in 2008. A total of 63 participants from these camps used the mobile telephone food record for meal session 1, and 55 (87%) returned for meal session 2 the following day. After using the mobile telephone food record for meal session 1, participants provided feedback and received additional training during the postmeal 1 session. During this session, the participants responded to a series of statements regarding their perceptions of the mobile telephone food record and preferences when using the mobile telephone food record. The advanced interactive instruction included activities in which the participants practiced taking images in potentially problematic snacking scenarios.

**Figure 1:**
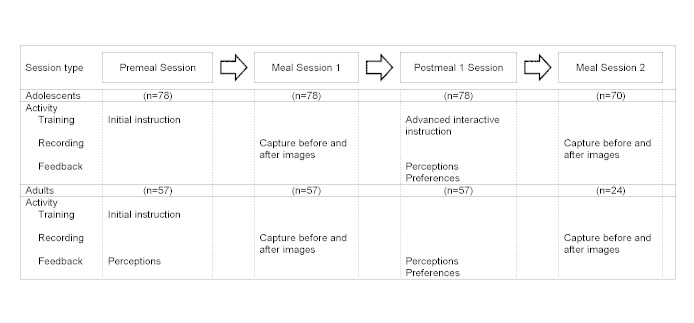
Study design, activities, and measures of participants using the mobile telephone food record. For 15 of the adolescent participants, meal session 2 was later in the same day. For the remainder of participants, meal session 2 occurred on a different day. Adult participants were offered dessert as a separate course. For meal session 1, 39 selected dessert, and for meal session 2, 15 selected dessert.

The second adolescent sample was a convenience sample drawn from the local community [31]. A total of 15 participants, ages 11–18 years, received all meals and snacks for a 24-hour period while being monitored under controlled conditions. These participants also took part in the feedback and advanced interactive instruction session after using the mobile telephone food record for meal session 1. Data from their first two meal sessions during the 24-hour period are included in this analysis. Figure 1 shows the data collection flow for the two samples of adolescents.

The adult sample was a convenience sample drawn from the campus of Purdue University and the local community during the fall of 2008. A total of 57 participants, ages 21–65 years, used the mobile telephone food record for meal session 1, and 24 (42%) returned for meal session 2 on a subsequent day (Figure 1). During the premeal session, the participants provided feedback regarding their perceptions of the mobile telephone food record, as well as their current use of mobile telephones and digital cameras, by responding to a series of statements and questions. After using the mobile telephone food record in meal session 1, the participants provided additional feedback during the postmeal 1 session, during which they responded to a series of statements regarding their perceptions of the mobile telephone food record and preferences when using the mobile telephone food record.

### Meal Sessions

**Figure 2:**
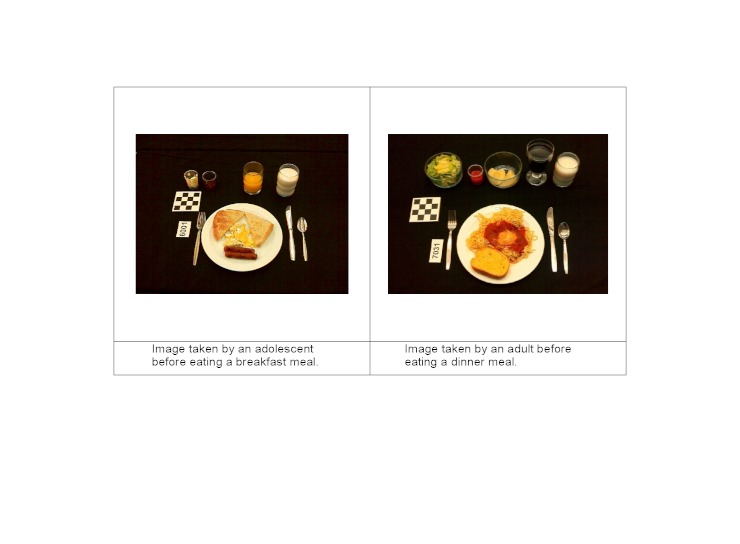
Images that demonstrate meeting two skills required for using the mobile telephone food record: included in the image are all foods and beverages and the entire fiducial marker (checkerboard square).

The menus served to the adolescents have been described previously [27]. For the adults, one breakfast menu and four dinner menus were cycled between the sessions. Figure 2 shows examples of meals served to adults and adolescents. Participants received instruction for using the mobile telephone food record during the premeal session (Figure 1). Use of the mobile telephone food record involves recording images of a meal before and after eating. Participants were instructed to include two items in each image: (1) all food and beverage, and (2) the entire fiducial marker (Figure 2). A fiducial marker is an object of known dimensions and markings, which serves as a size reference and must be included in the image [27]. The only instruction provided to participants for placement of the fiducial marker was to avoid placing it near beverages to prevent damage to the object. The meal environment was set up to mimic a restaurant dining atmosphere; however, participants were instructed not to mix or share their foods. The participants took an image of their meal prior to eating, saved the image, took an image of their meal after eating, and saved the image. Participants ate to satiation and, if they requested more, were served a second meal. At three of the four adult dinner meals, dessert was offered as a separate course. The process of capturing images was repeated for these desserts and any additional portions served.

We used HTC p4351 mobile telephones (HTC Corporation, Taoyuan, Taiwan) running Windows Mobile 6.0 Professional (2007; Microsoft Corporation, Redmond, WA, USA). The software, described previously [28,29], guided the user to select the meal occasion and capture an image of foods and beverages. After capturing the image, the user was prompted to review the image and was then given a choice to either retake or save the image. Once the user was satisfied with the image, the mobile telephone prompted the user to eat before proceeding to the next screen. At the next screen, the user was prompted to take an image of the place setting regardless of whether foods and beverages remained. The final screen showed the before and after images prior to exiting the program. If questions arose, the participants were assisted during meals by trained nutrition students. These students also recorded the number of image-capturing attempts before and after meals, as well as the number of images taken by each participant before capturing a satisfactory image and whether participants sat or stood to take images. Participants in the first adolescent sample were compensated US $5 per meal. The second adolescent sample participated for a 24-hour period and they were compensated US $85 for their time. Participants in the adult sample were compensated US $5 for the first meal session and US $15 for the second meal session.

### Image Evaluation: Skill Set

To assess the two skills of including all foods and beverages and the entire fiducial marker in the image, the before and after meal images were evaluated for the inclusion of these two required items. When evaluating the inclusion of all foods and beverages, the images were coded as yes if all of the foods and beverages were visible in the image, no if any of the food or beverage was not visible, or software programming error if the image was unavailable due to software malfunction. When evaluating for the inclusion of the entire fiducial marker, the images were coded as yes if the entire fiducial marker was visible in the image, no if a portion of the fiducial marker was cut off, or software programming error. To evaluate the skill of efficiently taking only one image, the number of images taken by each participant before and after meal sessions was coded as one image or greater than one image.

### Feedback Session: Perceptions and Preferences

During the feedback sessions, we showed statements regarding possible perceptions of the mobile telephone food record and preferences when using the mobile telephone food record using PowerPoint (Office 2007, PowerPoint 2007; Microsoft Corporation). The participants responded to these statements using a 5-category ordinal response scale (ie, strongly agree, agree, neutral, disagree, and strongly disagree).

We showed the following five statements regarding perceptions to all participants: (1) I think it would be easy to remember to take an image before meals, (2) I think it would be easy to remember to take an image after meals, (3) I think it would be easy to remember to take an image before snacks, (4) I think it would be easy to remember to take an image after snacks, and (5) the software was easy to use.

We showed the following four statements regarding preferences to all participants: (1) I think it would be easy to carry and use a credit card-sized fiducial marker, (2) I think it would be easy to carry and use a USB-sized fiducial marker (to denote size, this was defined to participants as USB flash drive, USB memory stick, USB jump drive, or USB thumb drive), (3) I prefer to stand while taking an image, and (4) I prefer to sit while taking an image.

The adolescents responded to these nine statements at the start of the postmeal 1 session, followed by the advanced interactive instruction. The adolescents’ responses were collected with the *e*Instruction Classroom Performance System (*e*Instruction, Cincinnati, OH, USA).

During the premeal session, participants in the adult sample responded to perception statements 1–4. We asked the adults questions to assess their previous experience capturing images with digital cameras and mobile telephones. These were (1) Do you own a digital camera? (2) How often have you taken pictures with a digital camera? and (3) How often have you taken pictures with a mobile telephone?

The response choices for these latter questions were frequently, occasionally, and never or rarely. The adult participants responded to the nine statements above in the postmeal 1 session. The adults recorded their responses on a paper form.

### Statistical Analysis

We used data that we collected using the same methods among the adults and the adolescents for statistical comparisons. To further delineate differences by age, we divided the adolescent sample into early and late adolescence: 11–14 years and 15–18 years, respectively. The adult sample was divided into early and middle adulthood: 21–40 years and 41–65 years, respectively. Descriptive analysis included frequencies and percentages. Within both the adolescent and the adult samples, we analyzed differences in age groups and gender using chi-square. McNemar test was used for comparisons of the set of skills for capturing images within the adolescents and within the adults. For those comparisons, each skill (eg, all foods being in image) was classified as yes (demonstrating the skill) or no (not demonstrating the skill). Chi-square was used for comparisons of the skill set between the adults and adolescents; for these comparisons, we grouped no and software programming error together. The 5-category ordinal response scales used by the participants to provide their preferences and perceptions were recoded as agree, neutral, or disagree. We compared perceptions and preferences between adults and adolescents using chi-square. For comparisons with an expected cell count of less than 5, limiting the comparison to agree and disagree eliminated the inadequate cell counts. We used SPSS 17.0 (IBM Corporation, Somers, NY, USA) for all statistical analyses.

## Results

A total of 135 participants (78 adolescents, 57 adults) used the mobile telephone food record for meal session 1, and 94 (70 adolescents, 24 adults) returned to use the mobile telephone food record for meal session 2. The descriptive characteristics of both samples are in Table 1. The average meal duration was 14 minutes for adolescents and 20 minutes for adults. The participants were of diverse ethnic backgrounds. Among the adults, 87% (39/45) claimed to own a digital camera and almost half (22/45, 49%) frequently used it to take pictures. All of the adult participants owned a mobile telephone, but only 16% (7/45) frequently took pictures with their mobile telephone.

Software programming errors occurred when saving the image on the mobile telephone food record, making them unavailable for the analysis. These errors resulted in partial loss of images, either a before or an after image; however, no images were available for only one adult participant, leaving 56 adults for this analysis. Changes to the software were made after testing it with the adolescents, which likely accounted for the reduction in programming errors experienced by the adults. Table 2 shows an evaluation of the participants’ ability to follow a defined set of skills when capturing images with the mobile telephone food record. The majority of adults (53/56, 95%) were able to include all foods and beverages in both the before and after images for meal session 1, while 96% (23/24) were able to do the same for meal session 2. A statistically significantly lower proportion of adolescents than adults were able to include all foods and beverages in both the before and after images for meal session 1 (*P *= .008). This proportion improved to being similar to that of the adults for meal session 2, as Table 2 shows.

For both adults and adolescents, inclusion of the fiducial marker in the image was more problematic than inclusion of all of the foods and beverages (Table 2). There were no significant differences between the adolescents and the adults. Among the adult participants self-selecting desserts, the inclusion of all the dessert and the fiducial marker was very high (Table 2). A significantly higher proportion of adults than of adolescents had to capture more than one image before (*P *= .03) and after (*P *= .008) meal session 1 to obtain an image suitable for image analysis (Table 3). This was also the case before and after meal session 2 between adults and adolescents, although this difference was not statistically significant. The adolescents significantly improved their efficiency with capturing suitable images from meal session 1 to meal session 2 (*P *= .04).

Table 4 shows perceptions of the mobile telephone food record and preferences when using the mobile telephone food record. The majority of both age groups (52/57, 91% of adults; 55/78, 71% of adolescents) agreed that the software was easy to use. Although the adults needed to take more images, they still perceived that capturing images with the mobile telephone food record was easy; however, the proportion was not significantly different from that of the adolescents. Compared with adults, adolescents were less likely to agree that it would be easy to take images before and after meals (*P *< .001).

Adolescents had a stronger preference than adults for the size of the fiducial marker that they would be willing to use (Table 4). The majority of adolescents (55/71, 77%) and adults (52/57, 91%) reported being willing to use a credit card-sized fiducial marker, but adolescents were less likely to prefer a USB-sized fiducial marker (*P *= .002). Adolescents reported they would prefer to stand while taking images (*P *< .001) while adults preferred to sit (*P *= .002) while taking images. For all analyses regarding skills, preferences, and perceptions, there were no significant differences by gender, early adolescence and middle adolescence, or early adulthood and middle adulthood.

**Table 1:** Characteristics of adults and adolescents testing the usability of the mobile telephone food record.

Characteristic		Adolescents (n = 78), n (%)	Adults (n = 57) n (%)
**Gender**			
	Male	26 (33%)	18 (32%)
	Female	52 (67%)	39 (68%)
**Age group (years)**			
	11–14	45 (58%)	NA^a^
	15–18	33 (42%)	NA
	21–40	NA	27 (47%)
	41–65	NA	30 (53%)
**Ethnic group**			
	Asian	1 (1%)	4 (7%)
	Hispanic	7 (9%)	0 (0%)
	Non-Hispanic white	55 (70%)	45 (79%)
	Black/African American	10 (13%)	2 (4%)
	Multiple	5 (6%)	6 (11%)

^a ^Not applicable.

**Table 2:** Evaluation of participants’ set of skills when capturing images with the mobile telephone food record.

Skill		Adolescents (n = 78)			Adults (n = 56)^a^		
		Yes, n (%)	No, n (%)	Software error^b^, n (%)	Yes n (%)	No, n (%)	Software error, n (%)
**All foods and beverages included in image**							
	Meal session 1^c^	61 (78%)	7 (9%)	10 (13%)	53 (95%)	0 (0%)	3 (5%)
	Meal session 2	59 (84%)	9 (13%)	2 (3%)	23 (96%)	0 (0%)	1 (4%)
	Dessert session 1^d^	NA^e^	NA	NA	39 (100%)	0 (0%)	0 (0%)
	Dessert session 2^d^	NA	NA	NA	14 (93%)	0 (0%)	1 (7%)
**Entire fiducial marker included in image**							
	Meal session 1	54 (69%)	14 (18%)	10 (13%)	44 (79%)	9 (16%)	3 (5%)
	Meal session 2	53 (76%)	15 (21%)	2 (3%)	18 (75%)	5 (21%)	1 (4%)
	Dessert session 1^d^	NA	NA	NA	37 (95%)	2 (5%)	0 (0%)
	Dessert session 2^d^	NA	NA	NA	11 (73%)	3 (20%)	1 (7%)

^a ^Due to software programming error, n = 56 instead of 57.

^b ^Paired images unavailable due to software programming errors.

^c^ *P *= .008 using chi-square and comparing adolescents versus adults.

^d ^Dessert was served as a separate course for adult participants. For meal session 1, 39 selected dessert, and for meal session 2, 15 selected dessert.

^e ^Not applicable.

**Table 3:** Comparisons between and within adolescents and adults of the number of images acquired prior to obtaining a suitable image.

Group			Adolescents (n = 63 meal session 1, n = 55 meal session 2)				Adults (n = 56^a ^meal session 1, n = 24 meal session 2)		
			1 image, n (%)	>1 image, n (%)	Data recording error^b^, n		1 image, n (%)	>1 image, n (%)	Data recording error^b^, n
**All participants**									
	Meal session 1								
		Before image^c,d^	38 (62%)	23 (38%)		2	21 (42%)	29 (58%)	6
		After image^c,e^	44 (75%)	15 (25%)		4	25 (50%)	25 (50%)	6
	Meal session 2								
		Before image	39 (77%)	12 (24%)		4	13 (59%)	9 (41%)	2
		After image	40 (78%)	11 (22%)		4	16 (73%)	6 (27%)	0
**Matched participants**^f^									
	Meal session 1								
		Before image	28 (58%)	20 (42%)^g,h^		NA^i^	9 (45%)	11 (55%)	NA
		After image^c,j^	36 (75%)	12 (25%)		NA	7 (35%)	13 (65%)	NA
	Meal session 2								
		Before image	38 (79%)	10 (21%)^g,h^		NA	12 (60%)	8 (40%)	NA
		After image	37 (77%)	11 (23%)		NA	14 (70%)	6 (30%)	NA

^a ^Due to software programming errors, n = 56 instead of 57.

^b ^Data recording error on the part of staff; therefore, numbers not included in percentages, which represent only users’ abilities.

^c ^Comparison between adolescents and adults.

^d^ *P *= .03.

^e^ *P *= .008.

^f ^Number of before and after meal images these participants took was recorded for both meal session 1 and meal session 2 (n = 48 session pairs for adolescents; n = 20 session pairs for adults).

^g ^Comparison between meal session 1 (before) and meal session 2 (before) within adolescents.

^h^ *P* = .04

^i ^Not applicable.

^j^ *P *= .002.

**Table 4:** Comparison of perceptions and preferences between adolescents and adults regarding use of the mobile telephone food record^a^.

Perceptions and preferences		Adolescents (n = 78)^b^			Adults (n = 57)		
		Agree, n (%)	Neutral, n (%)	Disagree, n (%)	Agree, n (%)	Neutral, n (%)	Disagree, n (%)
**Perceptions**							
	The software was easy to use	55 (71%)	9 (13%)	6 (9%)	52 (91%)	1 (2%)	4 (7%)
	I think it would be easy to remember to take an image before meals^c^	26 (37%)	22 (31%)	22 (31%)	47 (83%)	5 (9%)	5 (9%)
	I think it would be easy to remember to take an image after meals^c^	29 (41%)	27 (38%)	15 (21%)	42 (74%)	8 (14%)	7 (12%)
	I think it would be easy to remember to take an image before snacks	8 (11%)	16 (23%)	46 (66%)	15 (26%)	12 (21%)	30 (53%)
	I think it would be easy to remember to take an image after snacks	15 (21%)	19 (27%)	37 (52%)	19 (33%)	13 (23%)	25 (44%)
**Preferences**							
	I think it would be easy to carry and use a credit card-sized fiducial marker	55 (77%)	10 (14%)	6 (8%)	52 (91%)	4 (7%)	1 (2%)
	I think it would be easy to carry and use a USB-sized fiducial marker^d^	30 (42%)	19 (27%)	22 (31%)	38 (67%)	15 (26%)	4 (7%)
	I prefer to stand while taking an image^c^	43 (63%)	14 (21%)	11 (16%)	13 (23%)	12 (21%)	32 (56%)
	I prefer to sit while taking an image^d^	25 (36%)	21 (30%)	23 (33%)	39 (68%)	8 (14%)	10 (18%)

^a ^Percentages do not add to 100% due to rounding.

^b ^Missing values due to a malfunction of the *e*Instruction Classroom Performance System.

^c^ *P *< .001 using chi-square and comparing adolescents versus adults.

^d^ *P *= .002 using chi-square and comparing adolescents versus adults.

## Discussion

This is the first study to systematically evaluate the abilities of adolescents and adults to provide accurate images of an eating occasion. A priori, we assumed that huge differences in skills with technology between adolescents and adults would emerge; however, other than number of images captured, nothing else became obvious. The adolescents were more efficient: they took fewer images than the adults. By the second meal, the adolescents became even more efficient, whereas the adults made insignificant gains. Also, by the second meal, the inclusion of all foods in the images was the same between adults and adolescents, whereas inclusion of the important nonedible item (ie, the fiducial marker) was more problematic for both adolescents and adults. These results support that the fiducial marker was too large. As such, it was difficult to include in images without being partially covered by a plate or utensil. Evaluation of the images for the placement of the fiducial marker revealed that the participants placed the fiducial marker in various locations in the meal setting. Thus, work to reduce the size of the fiducial marker is justified. For both age groups, a notification from the device that the entire fiducial marker is not in the camera’s field of view may be helpful in reminding participants to include the entire fiducial marker when capturing images. Clear instruction on the desired placement of the fiducial marker may prevent the participant from spending time deciding where to locate it in the meal setting, which might reduce the burden of this task and translate to better cooperation with this step.

We have also established that the perceptions and preferences of adolescents and adults regarding use of the mobile telephone food record were more disparate than their skill set. In particular, adolescents were less likely than adults to agree that capturing images of meals before and after would be easy. Adolescents were more opinionated about preferring a credit card-sized fiducial marker. The adolescents may have preferred a credit card-sized fiducial marker because it could be easily carried in a wallet. Finally, adolescents stated a preference to stand while using the mobile telephone food record and adults preferred sitting. This preference for standing is consistent with irregular eating patterns and selecting snacks that are easily portable and often eaten while standing [32,33].

Adolescents are typically the earlier and more eager adopters of new technology [17]. Previous dietary assessment research on adolescents showed that they preferred methods using technology over typical paper or pencil methods [32]. In the current study, adults were noticeably less confident than the adolescents in using this new technology. Whereas the adolescents were eager to use the mobile telephone food record and quickly started taking images, the adults were much more cautious and asked more questions prior to taking an image. This could explain the adults being more likely to include all foods and beverages in the image. However, the adolescents’ skills matched the adults’ after extensive training, a phenomenon previously documented by Six and colleagues [27]. In all cases, it is impossible to separate the participants’ skill in using the mobile telephone food record from motivation to follow the instructions given.

Despite the adolescents being observed as more confident and comfortable when using the mobile telephone food record, they were less likely to agree that it was easy to capture pre and post meal images. This could be a result of differences in daily schedules between the two age groups and may reflect adolescents having more irregular meal times than adults [14,15]. Alternatively, the adolescents may have higher expectations of and demands from the technologies they use [17].Therefore, for adolescents, improvements to the mobile telephone food record might include more reminders throughout the day to ensure that they capture both before and after meal images. For all users, a reminder system, such as an alarm or pop-up message, will likely be needed to remind participants to record their snacks.

Based on the length of time between the before and after images, the average meal duration was shorter for adolescents than for adults. This information provides a basis for programming age-specific software to start timing after the first meal image is captured to initiate a reminder for taking the after image. Next steps include testing the mobile telephone food record with participants in a free-living environment to ascertain the true level of burden, duration of cooperation, and accuracy of recorded energy intake using a biomarker for energy, such as doubly labeled water. There were minimal differences regarding preferences, which will simplify the design process for the mobile telephone food record for adults and adolescents.

### Conclusions

The results of these studies will translate to minimal design differences of the mobile telephone food record between adolescents and adults. The majority of both adults and adolescents were able to follow the defined set of skills when capturing before and after images of their meals; however, these results do provide evidence for the need for some age-specific development of the mobile telephone food record, such as reminder programming. The adults were more cautious than the adolescents when taking images and as a result were more likely to include all food and the fiducial marker, which are necessary to capture an image suitable for image analysis. However, adults had to take more images than adolescents before capturing satisfactory ones. Although they were less efficient, the adults perceived that remembering to capture images with the mobile telephone food record would be easy. Additional use of the mobile telephone food record improved adolescents’ perceptions and set of skills when capturing images. Additional interactive training will likely be necessary for all users to provide extra practice in taking images before entering a free-living situation. The adolescents had a stronger opinion about the size of the fiducial marker than the adults, suggesting that the fiducial marker design needs to accommodate adolescents over adults. Software improvements between the adolescent and adult meal sessions greatly reduced the number of software programming errors. Some problems will likely never be entirely eliminated due to low battery power and other software-related difficulties, but advances in technology will ensure that these errors will become less frequent.

A more accurate method of dietary assessment will help strengthen the ability of researchers to identify diet–disease and diet–gene relationships. The data generated from a tool such as the mobile telephone food record could be combined with measures of the built environment to inform public policy and assist in the development of nutrition interventions. Further, novel dietary assessment methods will contribute to the growth of mobile applications to enhance self-monitoring for diabetes, weight control, and other diet-related diseases.

## Abbreviations

mpFR: mobile telephone food record
PDA: personal digital assistant
